# Are Small Cities More Environmentally Friendly? An Empirical Study from China

**DOI:** 10.3390/ijerph16050727

**Published:** 2019-02-28

**Authors:** Shuai Liu, Fei Fan, Jianqing Zhang

**Affiliations:** 1 Institute of Central China Development, Wuhan University, Wuhan 430072, China; liushuai@whu.edu.cn; 2 Division of Economics, School of Social Sciences, Nanyang Technological University, Singapore 637332, Singapore; 3 School of Earth and Environmental Sciences, The University of Queensland, Brisbane 4072, Australia; 4 School of Economics and Management, Wuhan University, Wuhan 430072, China

**Keywords:** PM_2.5_, city size, STIRPAT, China

## Abstract

City sizes are rapidly expanding, and urban air pollution is a serious challenge in China. PM_2.5_ (fine particulate matter) is the primary pollutant of urban pollution. This study aimed to examine the correlations between PM_2.5_ and city size. In this paper, using the panel data of 278 cities in China from 2007 to 2016, we constructed a static and dynamic panel model based on the STIRPAT (Stochastic Impacts by Regression on Population, Affluence and Technology) analytical framework. We found that there was a significantly inverted N-shaped correlation between PM_2.5_ and city size. Two inflection points were found at 949,200 and 3,736,100. We found no evidence to support the EKC (Environmental Kuznets Curve) hypothesis, while the “Pollution Haven Hypothesis” gained support. The contradiction between PM_2.5_ and city size will exist for the long term. Policy recommendations were proposed based on our findings. Controlling the city size does not seem to be necessary for very large cities as they have passed the second inflection point. Cities with a growing population are under great pressure to prevent PM_2.5_ pollution and need to implement greater measures to reduce pollution.

## 1. Introduction

China’s urbanization ratio (measured by population) increased from 44.9% in 2007 to 58.52% in 2017, as the size of Chinese cities has grown rapidly. The number of cities with an urban population over 1 million increased from 57 in 2007 to 75 in 2016. Beijing and Shanghai have become megacities, whose populations have reached 18.79 million and 24.19 million, respectively. The continuous expansion of the city size has caused “urban diseases”, of which pollution has become the first and foremost. Han [1] argued that the rapidly growing urbanized population in China has caused serious environmental pollution, particularly air pollution. Urban pollution is closely linked to the size of the city—as the size of the city increases, the total amount of urban pollution emissions increases. Therefore, the expansion of cities has created serious urban pollution and placed tremendous pressure on the environment. Urban pollution leads to economic losses and threatens the climate and human health. Xie et al. [2] used a CGE (Computable General Equilibrium) model, a class of economic models that use actual economic data to estimate how an economy might react to changes, to estimate economic losses, where the results showed that without control measures, China will experience a 2.00% loss in gross domestic product (GDP) and a 25.2 billion USD increase in health expenditure from PM_2.5_ (fine particulate matter) pollution in 2030. Tai [3] found that there was a strong positive correlation between PM_2.5_ components and temperature across most of the USA. Studies have shown that there is a causal relationship between PM_2.5_ exposure and cardiovascular morbidity and mortality [4]. A reduction in exposure to ambient fine-particulate air pollution has contributed to significant and measurable improvements in life expectancy in the United States [5].

Air pollution has increasingly attracted public attention in China. In 2015, more than 99% of deaths due to household air pollution and approximately 89% of deaths due to ambient air pollution occurred in low-income and middle-income countries, and more than 50% of global deaths caused by ambient air pollution in 2015 occurred in India and China [6]. The Chinese government has acknowledged the dangers posed by pollution and has set specific targets for environmental improvement and restrictions on the use of certain resources. China has implemented a vast network of stations to monitor air quality in more than 400 cities. The capacity to track emissions has been central to developing policy and to implementing data-driven regulatory frameworks. As a result, China has increased its reliance on non-fossil energy sources (predominantly renewables and nuclear) from 9.4% of total energy use in 2010 to 12.0% in 2015, surpassing the 12th Five-Year Plan target of 11.4% by 2015. The most recent Five-Year Plan aims to increase non-fossil energy use to at least 15% by 2020, and to at least 20% by 2030.

**Figure 1:**
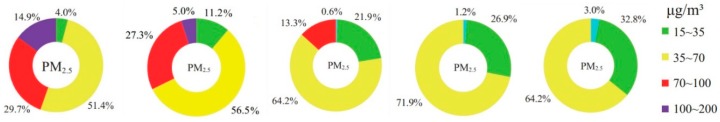
Proportion of cities in China with varying PM_2.5_ (fine particulate matter) concentrations: 2013–2017.

Although many preventive measures have been undertaken, the current condition of air pollution in China is still undeniably serious. In 2017, among the 338 prefecture-level (and above) cities, only 99 cities were reported to meet the environmental air quality standards, accounting for only 29.3% of the total number of cities. Moreover, the air quality of 239 cities was below standard, accounting for 70.7%. According to the National Ambient Air Quality Standards set by the U.S. EPA (Environmental Protection Agency), the PM_2.5_ standard is 35 μg/m^3^. As depicted in Figure 1, in recent years, more than 70% of cities in China have not met the environmental standards.

Many cities have set out clear plans regarding city size limits in China. For example, from the perspective of ecological capacity (available water resources and per capita water resources), Beijing has determined that the size of its urban population should be under 23 million in the long term. According to the City Master Plan, many other cities have proposed controlling their city size (measured by population) such as Shanghai, Guangzhou, Shenzhen, and Wuhan. Controlling the size of the city seems to have become an important means of managing “urban diseases”. However, from another perspective, the expansion of city size is conducive to the formation of the urban economy of scale. The high concentration of the population has led to the intensive use of energy and an efficient reduction in pollution emissions, which has resulted in a gradual decline in per capita pollution. However, limiting the city size may not decrease the urban pollution.

City size is an important topic in urban economics research and is usually measured by urban population. The optimal city size is reached when the urban marginal costs equal the marginal benefits. Camagni et al. [7] formulated an equilibrium urban size model and generated a model using 59 European cities, concluding that there was no single optimal city size as each city had its own “equilibrium” size. Duan [8] used the cross-sectional data of 284 prefecture-level cities to study the optimal city size. On the basis of quantile regression, the city size was shown to be affected by factors such as market size, public finance, knowledge spillover, and urban–rural income gap. Wang [9] reviewed China’s urbanization process and explored the optimal size and urbanization path of Chinese cities, which showed that the city size was related to the economic development level, traffic conditions, and geographical location. Pollution is another important topic. The Environmental Kuznets Curve (EKC) proposed by Grossman and Krueger [10] explains the coordinated development of economic and environmental degradation. The EKC hypothesis argues that there exists an inverted U-shape between economic development and pollution, implying that environment pollution increases with economic output at an early stage, but decreases as economic output surpasses the inflection point. The EKC hypothesis has been validated in upper middle and high-income countries [11], where the existence of the inverted U-shaped relationship has been adapted for different environmental degradation factors, such as CO_2_ [12], SO_2_ [13], and waste water [14].

Some scholars have investigated the relationship between air pollution and city size; however, no consistent conclusions have been reached. Han et al. [15] stated that large cities had contributions of 5.40 ± 4.80 µg/m^3^·PM_2.5_ year per million people in China. Shukla and Parikh [16] focused on the relationship between ambient air quality and city size and studied the relationship theoretically and empirically using data from international cities. They found that a positive association between poor air quality and city size and developed–developing country differences emerged. Han [17] selected 350 prefectures in China to estimate the nexus between PM_2.5_ and city size using one-way analysis of variance (ANOVA) and Fisher’s least significant difference (LSD) and found that PM_2.5_ was significantly correlated with city size. Oliveira [18] found support of a power-law superlinear scaling behavior between CO_2_ emissions and city size using data from U.S. cities. Cole and Neumayer [19] found evidence that population increases were matched by proportional increases in carbon dioxide emissions and a U-shaped relationship for sulfur dioxide emissions. Crame [20] made an empirical case study based on a modified IPAT (Human Impact, Population, Affluence and Technology) model and found that a large population was associated with a greater increase in air pollutant emissions. Zhang [21] used panel data from 29 provinces spanning the period from 1997 to 2012 to demonstrate that population quality increased China’s carbon emissions. Liddle [22] utilized panel regressions based on 12 different time spans in 80 countries to evaluate the carbon emissions elasticity of the population. Liddle’s results indicated that elasticity was likely not robust or statistically significantly different from either an OECD (the Organisation for Economic Co-operation and Development) or a non-OECD country. Zhou and Liu [23] concluded that urbanization, represented by population, positively affected CO_2_, particularly in China’s eastern and central regions. U-shaped relationships [24] have also been detected, which suggests a complicated correlation between city size and the atmospheric environment.

Air pollution is the foremost form of urban pollution and has led to wide public concerns. Airborne particulate matter and ambient air pollution are proven group 1 human carcinogens [25]. Urban population size and its related activities have been confidently attributed to urban air pollution, and there is a significant correlation between air pollution and the population [26]. As the Chinese government is continually proposing new urbanization, it is bound to cause a further expansion of the city size. Will the expansion of city size inevitably lead to an increase in PM_2.5_ pollution? What is the relationship between PM_2.5_ pollution and city size? Can controlling the size of the city improve the quality of the urban environment? This paper aimed to answer these questions by examining the correlation between PM_2.5_ pollution and city size. The goal of this study was to achieve a win–win situation for both city size and PM_2.5_ pollution.

In general, the correlation between city size and air pollution has been widely studied based on different quantitative techniques, research areas, and variables. Several shortcomings still exist regarding the relationship between city size and air pollution. First, PM_2.5_ is the primary air pollutant of air pollution, but many previous studies have focused on other pollutants such as NO_x_ and SO_2_. Therefore, there has been little research in terms of PM_2.5_, and an inadequate understanding of the relationship between city size and PM_2.5_ still remains. Second, studies have seldom taken into account the nonlinear relationship and regional heterogeneity for country-level research. Third, both panel and cross-section data and approaches have been used, but the endogeneity problem has been neglected. Therefore, this paper attempts to fill these gaps. This paper collected unbalanced panel data from 278 cities in China. The long time span and high number of observation samples allowed us to obtain a robust conclusion in comparison with previous studies based on provincial data. On the basis of the extended STIRPAT (Stochastic Impacts by Regression on Population, Affluence and Technology) model, a classic useful analytic tool for disciplining environmental policy with an empirical foundation, this study built fixed panel models to estimate the correlations between city size and PM_2.5_, and dynamic panel models were used for checking the robustness.

The remainder of the paper is organized as follows. Section 2 presents the data description and model specification, which is followed by the empirical results and regional heterogeneity test in Section 3 and Section 4. Finally, Section 5 presents our conclusions.

## 2. Model and Data

### 2.1. Theoretical Framework

The IPAT model proposed by Ehrlich and Holdren [27] is a classic model for analyzing the impact of human activities on the environment. The basic equation of the model is *I* = *PAT*, where *I* represents pollution; *P* represents population; *A* represents the level of abundance (understood as per capita consumption or per capita GDP); and *T* refers to technology (understood as the impact of unit economic activity on pollution, so it can be expanded into multiple economic variables). The IPAT model has been successfully used to analyze sustainability assessments until recent years [28]. The IPAT model was widely adopted and extended into models like the Kaya equation [29], ImPACT [30], IPBAT [31], and ImPACTS [32]. However, this type of model has obvious shortcomings. First, it is based on the assumption that the change in pollution and the change of various influential factors occur at the same scale, that is, *P* (or *A*, *T*) doubles as well as environmental pollution. This clearly does not match reality. Second, the model is given in the form of accounting equations, so hypothesis testing cannot be performed. Third, the model cannot capture nonlinear relationships [33]. To overcome the shortcomings of the IPAT models, Dietz and Rosa developed these models into a stochastic model, that is, STIRPAT [34]:

(1) $$I_{i}=aP_{i}^{b}A_{i}^{c}T_{i}^{d}e_{i}.$$

Take the natural logarithm of the two sides of the equation:

(2) $$\ln I_{i}=\alpha +b\ln P_{i}+c\ln A_{i}+d\ln T_{i}+\varepsilon_{i},$$

where subscript i denotes the observational units; a represents a constant term and b, c, and d are coefficients; e represents an error term; and α and ε are the values of a and e, respectively, after taking the natural logarithm form. Dietz and Rosa also pointed out that P, A, and T could be decomposed, in addition to other reasonable control variables that could be added to the equation.

The STIRPAT model has been widely and successfully applied. For example, Shahbaz et al. [35] investigated the relationship between CO_2_ emissions and urbanization in Malaysia using the STIRPAT model and VECM (Vector Error Correction Model) Granger causality test. Chai et al. [36] adapted the STIRPAT model to forecast future natural gas consumption in China. In addition, Hua et al. [37] established a STIRPAT model and analyzed the effect of fiscal spending on air pollution in Chinese cities. The previous literature indicates that the STIRPAT model is flexible and can be refined using different functional forms. Thus, a STIRPAT model was used in this study to explain the city size-PM_2.5_ nexus.

### 2.2. Econometric Model

On the basis of the STIRPAT model, we set up the econometric model by expanding and decomposing the P, A, T variables. The basic static panel model was established as follows:

(3) $$\begin{array}{l} ln\;PM_{it}=\beta_{0}+\beta_{1}\;ln\;CZ_{it}+\beta_{2}(ln\;CZ_{it}){}^{2}+\beta_{3}(ln\;CZ_{it}){}^{3}+\beta_{4}\;ln\;PGDP_{it}\\ \;+\beta_{5}(ln\;PGDP_{it}){}^{2}+\beta_{6}\;ln\;EC_{it}+\beta_{7}\;ln\;ES_{it}+\beta_{8}\;ln\;TO_{it}\\ \;+\beta_{9}\;ln\;PE_{it}+{µ}_{i}+\lambda_{t}+\eta_{it} \end{array}$$

In addition to the basic static model, we set up a dynamic model for a robustness check. Equation (4) shows the dynamic panel model, which includes lagged PM_2.5_ as an independent variable. On the one hand, the basic static model is confronted with the endogeneity problem. The dynamic panel model can alleviate this problem by using lagged variables as instrument variables. On the other hand, current pollution may be influenced by past situations. It has been proven that PM_2.5_ pollution shows a snowball effect in the dimension of time [38].

(4) $$\begin{array}{l} ln\;PM_{it}=\beta_{0}+\alpha \;ln\;PM_{it-1+}+\beta_{1}\;ln\;CZ_{it}+\beta_{2}(ln\;CZ_{it}){}^{2}+\beta_{3}(ln\;CZ_{it}){}^{3}\\ \;+\beta_{4}\;ln\;PGDP_{it}+\beta_{5}(ln\;PGDP_{it}){}^{2}+\beta_{6}\;ln\;EC_{it}+\beta_{7}\;ln\;ES_{it}\\ \;+\beta_{8}\;ln\;TO_{it}+\beta_{9}\;ln\;PE_{it}+{µ}_{i}+\lambda_{t}+\eta_{it} \end{array}$$

where subscript i represents the cities and t is the time in years. $\beta_{0}$ is the constant, and $\eta_{it}$ is the error term. $\beta_{1}–\beta_{9}$ represent the slope coefficients. City fixed-effects $\mu_{i}$ and time fixed-effects $\lambda_{t}$ are also included.

First, city size (denoted by CZ) is the core explanatory variable. The primary indicator of city size is urban population, that is, the people living in cities and not in rural areas. To explore the nonlinear relationship between city size and PM_2.5_, we added three squared and cubic terms of city size into the econometric model.

Second, abundance is measured by per capita GDP (denoted by PGDP). To capture the inverted U-shaped relationship, the squared per capita GDP was added to the model.

Third, while Lin et al. [39] employed energy intensity to measure technology, Poumanyvong and Kaneko [40] indicated technology using the share of industry and service in GDP. Thus, we decomposed T into two variables—energy consumption (denoted by EC) and economic structure (denoted by ES). These are measured by the amount of liquefied petroleum gas supply and the ratio of secondary industry, respectively.

Finally, two control variables were introduced to our model: trade openness (denoted by TO) and public expenditure (denoted by PE). The “Pollution Haven Hypothesis” predicts that the liberalized trade in goods will lead to the relocation of pollution from developed countries to developing countries. The trade openness is measured by the amount of foreign capital actually utilized. Expansionary fiscal spending has validated an alleviation effect on CO_2_ emissions [41]. Public expenditure is an indispensable control variable.

### 2.3. Econometric Methodology

There are two estimator approaches for the static panel model; namely the fixed effects model and the random effects model. We selected the optimal model using the Hausman test. The null hypothesis of the Hausman test is that the difference in coefficients is not systematic. The Hausman test can be used to differentiate between fixed effects model and random effects model in panel data. In this case, random effects is preferred under the null hypothesis because of higher efficiency, while under the alternative, fixed effects is at least as consistent and thus preferred. If the null hypothesis is rejected, the fixed effects model is suitable. We conducted the Hausman test, and the results showed that the Chi-square p-value was 0.000, which means that the model rejects the null hypothesis at the 1% significance level. Thus, the fixed effects model was used to estimate the static panel model.

Regarding the dynamic panel model, the difference and system generalized methods of moments (DGMM and SGMM, respectively) can be used for the estimation. The DGMM is prone to the problem of weak instruments. The SGMM considers both difference and level equations as an equation system that can improve the estimation efficiency. This study adopted SGMM as the estimator approach for the dynamic panel model. Furthermore, SGMM requires the Hansen J test to ensure that the instruments are exogenous and the AR(2) test to examine no second-order serial correlation.

The curve shapes between PM_2.5_ and city size can be determined from the signs of coefficients $\beta_{1}$, $\beta_{2}$, and $\beta_{3}$, as shown in Table 1. To obtain a valid result, the model is estimated as follows: test $\beta_{1}$, $\beta_{2}$, and $\beta_{3}$ first; if $\beta_{3}$ is not significant (*p* > 0.1), then drop the third power of city size and reevaluate the model to examine the U-shape or inverted U-shape curve; if $\beta_{2}$ is not significant (*p* > 0.1), then eliminate the square city size and re-evaluate the model.

### 2.4. Data

The PM_2.5_ data in this paper were obtained from the Department of Physics and Atmospheric Science, Dalhousie University, Canada. Donkelaar et al. [42] estimated PM_2.5_ by combining aerosol optical depth (AOD) retrievals from NASA, MODIS, MISR, and Sea WIFS instruments with the GEOS-Chem chemical transport model, which were subsequently calibrated to global ground-based observations of PM_2.5_ using geographically weighted regression (GWR). We deflated per capita GDP data into comparable data from 2007. RMB is the official currency of the People’s Republic of China. We converted the amount of foreign investment into RMB using the annual average exchange rate of RMB for that year and then used the GDP deflator to deflate. Other variables related to price factors were also deflated by the GDP deflator. We removed the missing values, and subsequently assigned the value of 0 to the value of 1 to make its logarithm meaningful. Finally, we obtained the unbalanced panel data of 2389 observations in 278 cities from 2007 to 2016. The data description and source are shown in Table 2. Table 3 presents the descriptive statistics of the main variables.

Table 4 presents the correlation coefficients among the core variables, partly for checking the relationships among variables, especially the correlation between PM_2.5_ and city size. It is clear that the correlation coefficients between PM_2.5_ and city size were statistically significant at the 1% level.

## 3. Results

### 3.1. Baseline Results

To avoid the multicollinearity problem and examine the respective influences of each independent variable, we employed stepwise regression. The empirical results for Equation (3) are presented in Table 5. Column 1 includes only two core explanatory variables: city size and per capita GDP. Other variables were introduced into columns 2–5 in sequence.

Almost all of the coefficients were very significant. The F statistics were statistically significant at the 1% level, which indicates that the models were significant as a whole. On the basis of columns 1–5, the coefficient signs and significance of the city size and per capita GDP were invariant as other variables were added, showing that the results were robust. The added variables were also significant, especially in column 5. The variables selected in the model were all relevant to PM_2.5_.

The coefficient signs of city size ($\ln CZ,\;{\left(\ln CZ\right)}^{2},{\left(\ln CZ\right)}^{3}$) suggest that an inverted N-shaped curve nexus existed between PM_2.5_ and city size. All coefficients of city size were statistically significant at the 1% level, which demonstrates a robust inverted N-shaped curve. PM_2.5_ showed a significant negative response to the expansion of city size at the early stage. When the city size reached the first inflection point, it exerted a positive and significant effect on PM_2.5._ As the city size increased to the second inflection point, the effect was negative and significant. The first inflection point was 949,200 and the second was 3,736,100 as estimated based on column 5. According to the urban population statistics of 2016, 131 cities, accounting for 45.8% of 286 cities in China, had not reached the first inflection point; most of these cities are located in the central and western parts of China. Only 24 developed cities had passed the second inflection point. Therefore, city size had a positive and statistically significant impact on PM_2.5_ for the 131 cities.

The coefficients of per capita GDP were negative and significant at the 1% level, while its squared terms were significantly positive, reflecting a U-shaped relationship between PM_2.5_ and economic development, and rejecting the existence of an Environmental Kuznets Curve (EKC).

Energy consumption exerted a negative effect on PM_2.5_, but the coefficients were small and not significant in columns 3 and 4. Similarly, the economic structure and the share of secondary industry presented an insignificant negative effect on PM_2.5_. Energy consumption and economic structure seemed to affect PM_2.5_ weakly and insignificantly. This negative effect may be the result of the promotion of clean energy and economic structural transformation proposed by the Chinese government, especially after the 2008 financial crisis. Fiscal spending measured by public expenditure also had a negative impact on PM_2.5_ with a 10% significance level, which indicates that fiscal policy work aimed to alleviate PM_2.5_ as a 10% increase in public expenditure reduced PM_2.5_ by 0.34%. Regarding trade openness, a positive and significant (at the 5% level) effect on PM_2.5_ was observed, corresponding to the “Pollution Haven Hypothesis”. An increase in the amount of foreign investment by 10% would lead to an increase in PM_2.5_ by approximately 0.07%, which was weak but significant.

### 3.2. Robustness

#### 3.2.1. Period Results

We separated all of the data into two periods, 2007–2011 and 2012–2016. We employed the same method for the baseline results to estimate Equation (3). Table 6 and Table 7 present the empirical results of the two periods 2007–2011 and 2012–2016. The coefficient signs of city size ($\ln CZ,\;{\left(\ln CZ\right)}^{2},{\left(\ln CZ\right)}^{3}$) were in accordance with the baseline results. All coefficients of city size were statistically significant at least at the 10% level. The results of both periods demonstrate a robust inverted N-shaped curve between PM_2.5_ and city size.

#### 3.2.2. Dynamic Panel Model

The dynamic panel model was estimated using SGMM for the robustness test. Table 8 displays the empirical results of the dynamic model. The signs of the coefficients were almost in accordance with the static model. The AR(1) tests showed that the first-order series was correlated significantly, while the AR(2) tests did not reject the absence of second-order autocorrelation. As variables were added to the model, the *p*-values of the Hansen test increased obviously, which implied that there was no rejection of the hypothesis of the validity of lagged variables in the levels and in the difference of instruments. Thus, the results were effective and robust. It is notable that the coefficients of the lagged PM_2.5_ were positive and strongly significant. Consequently, the previous PM_2.5_ increasingly led to the aggravation of the current PM_2.5_.

Through comparison of the baseline results and the two robustness tests, periods, and dynamic panel mode, effective and robust conclusions were drawn. There exists an inverted N-shaped relationship between PM_2.5_ and city size. We found no evidence in support of the EKC hypothesis, while the “Pollution Haven Hypothesis” was validated.

## 4. Regional Heterogeneity

**Figure 2:**
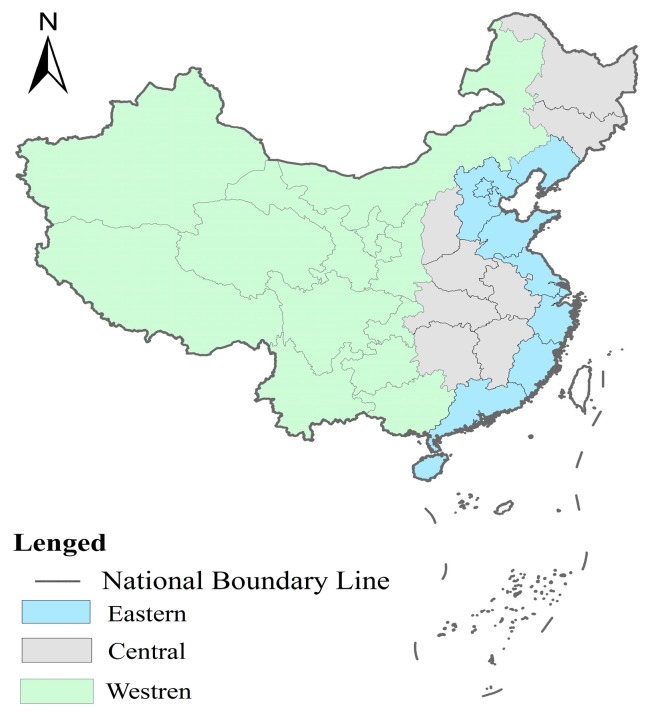
Distribution of the 30 provincial-level divisions in the three areas of China.

**Figure 3:**
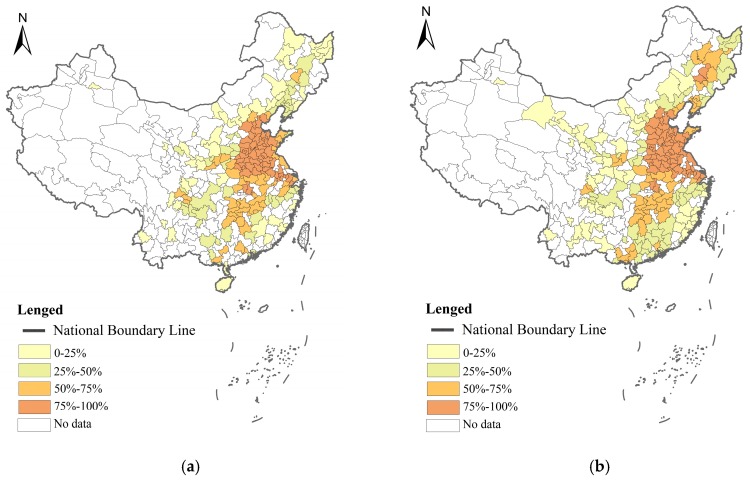
Cluster maps of PM_2.5_ concentrations in China. (**a**) Cluster map of PM_2.5_ concentrations in 2007; (**b**) cluster map of PM_2.5_ concentrations in 2016.

**Figure 4:**
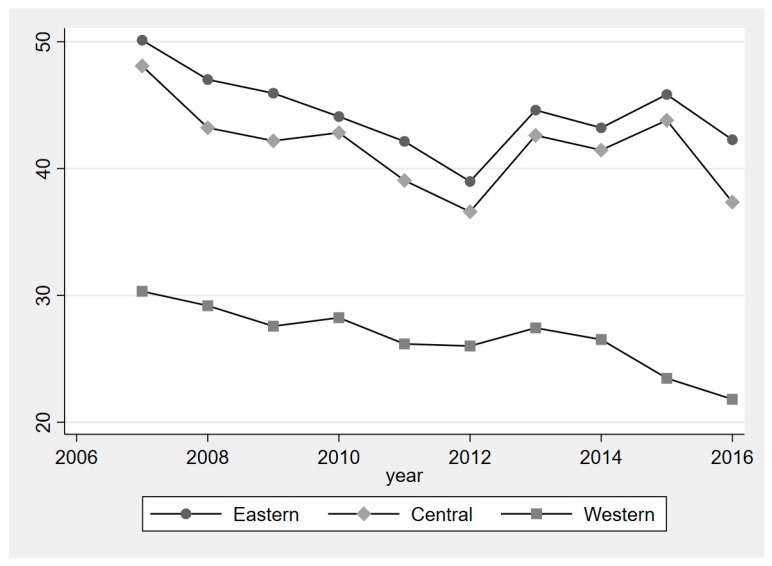
Average PM_2.5_ concentration between 2007 and 2016 in the three different regions.

China has a large territory with many differences between the regions. We divided the 30 provinces into three regions as per the official division method, which is mapped in Figure 2. Both PM_2.5_ and city size were different in the different regions. Cluster maps, showing the clusters and outliers of PM_2.5_ concentrations for 2007 and 2016, are given in Figure 3. Figure 4 displays the average PM_2.5_ concentration between 2007 and 2016 in the three different regions. The PM_2.5_ concentration is higher in eastern China than that in central and western China. Overall, the past 10 years has witnessed a decrease in the PM_2.5_ concentration. However, we can see quite different patterns in different regions. Although the PM_2.5_ concentration was higher than in central China, there were some similarities between eastern and central China in the changing trends of PM_2.5_ concentration. There were significant drops between 2007 and 2012, resulting in the lowest level in 2012. This was followed by a fluctuating increase until 2015. In 2016, there was a significant decline. In western China, the PM_2.5_ concentration experienced a steady decrease from 2007 to 2016.

**Figure 5:**
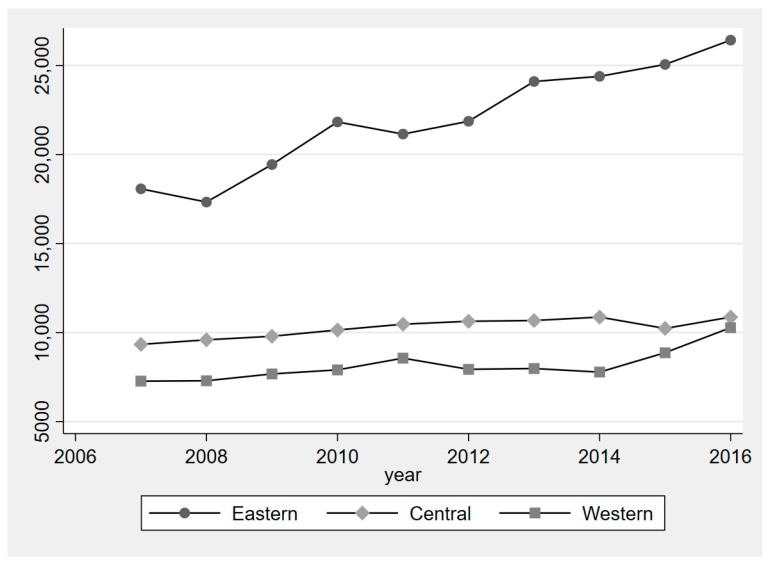
Urban population between 2007 and 2016 in three different regions.

As for city size, Figure 5 indicates the change in the urban population of the different regions. The city size in eastern China increased significantly and was larger than that in central and western China. The city size in central China was larger than that in western China; however, the urban population gap is closing all the time. Therefore, central and Western China have similarly witnessed a gradual increase in their urban population.

There were differences in both the PM_2.5_ concentration and city size among the three regions. In the National Main Functional Area Planning released by the Government of China, the land space in China has been divided into four areas: optimized development area, key development area, restricted development area, and prohibited development area. While most of the eastern and central regions belong to the optimized and key development areas, most of western China belongs to restricted and prohibited development areas.

PM_2.5_ pollution in China has been proven to have regional differences [43]. Wu [44] found that the relationship between PM_2.5_ and population in China was complex because of regional differences. Thus, we examined whether regional differences existed for the relationship between PM_2.5_ and city size in a territory as vast as China.

The regional empirical results are shown in Table 9. The F statistics were statistically significant at the 1% level, indicating that the three models were suitable and significant. There were differences in different regions, for example, in eastern China, an inverted N-shaped curve existed between PM_2.5_ and city size that was significant at the 10% level and was in line with all sample regressions (shown in column 5, Table 3). However, the relationship in central and western China was different. The results demonstrated a U-shaped curve relationship and a negative linear relationship in central and western China, respectively. In western China, the coefficients of per capita GDP were consistent for all cities, which negates the EKC hypothesis. The coefficients of per capita GDP in eastern and central China were contrary to those in western China at an insignificant level.

For the other controlled variables, energy consumption exhibited a negative impact on PM_2.5_, which was similar throughout China, with different levels of significance. While the economic structure exerted a significant and negative effect on PM_2.5_ in eastern and central China, a positive and significant effect was observed in western China. The effects of trade openness were positive but weak in all three regions. Public expenditure presented a negative and significant effect only for eastern China, with weak effects for central and western China.

## 5. Conclusions and Implications

This study investigated the relationship between PM_2.5_ and city size in China. PM_2.5_ pollution leads to serious public concerns. In this study, we provided city-level evidence of the effect of city size on PM_2.5_ pollutants. This paper applied the theoretical and analytical framework of the STIRPAT model to a static panel model using 278 cities between 2007 and 2016, and the dynamic models were built for the robustness check. Regional differences were also examined. Our conclusions are as follows:

(1) There was a significantly inverted N-shaped correlation between PM_2.5_ and city size. According to the estimation, the two inflection points were 949,200 and 3,736,100.
(2) We found no evidence to support the EKC hypothesis, while the “Pollution Haven Hypothesis” was proven. Energy consumption, the share of second industry, and public expenditure all presented a negative and significant effect on PM_2.5_.
(3) We found that regional differences did exist. In Eastern China, the N-shaped correlation between PM_2.5_ and city size was in accordance throughout China. The EKC hypothesis has still not been validated. The regional regressions showed a U-shaped curve relationship and negative linear relationship in central and western China. In different regions, the coefficient signs and significance of energy consumption, the share of second industry, and public expenditure were heterogeneous and complicated.

On the basis of our findings, we propose the following policy recommendations to facilitate a more environmental city.

(1) The positive correlation between PM_2.5_ and city size will exist for most cities for the long term. Only 24 developed cities, most of them located in eastern China, have passed the second inflection point. Almost every city is facing or will face the situation of increasing PM_2.5_ as the size of the city expands. To this end, policy makers in cities with a growing population need to implement greater measures to reduce pollution.
(2) The inverted N-shaped correlation suggests that controlling the city size does not seem to be necessary for very small and very large cities. PM_2.5_ did not increase with the size of the city for very large cities, possibly because an efficient reduction in pollution emissions has been reached in large cities as a result of technological progress.
(3) The effect of trade openness seems to suggest that international firms are more environmentally friendly. Policy makers may consider taking measures to attract more FDI (Foreign Direct Investment) to reduce pollution.
(4) The different estimation results for eastern, western, and central China suggest that it is necessary for local governments to have more flexibility in designing policies on environmental issues.

## Figures and Tables

**Table 1:** Curve shapes and the signs of coefficients.

The Signs of Coefficients	Curve Shapes
$\beta_{1}=\beta_{2}=\beta_{3}=0$	No relevance
$\beta_{1}\neq 0,\;\beta_{2}=\beta_{3}=0$	Monotonically increasing ($\beta_{1}>0$) linear relationship
	Monotonically decreasing ($\beta_{1}<0$) linear relationship
$\beta_{1}<0,\;\beta_{2}>0,\beta_{3}=0$	U-shape
$\beta_{1}>0,\;\beta_{2}<0,\beta_{3}=0$	Inverted U-shape
$\beta_{1}>0,\;\beta_{2}<0,\beta_{3}>0$	N-shape
$\beta_{1}<0,\;\beta_{2}>0,\beta_{3}<0$	Inverted N-shape

**Table 2:** Data description and source. GDP—gross domestic product.

Variables	Definition	Description	Source
PM	PM_2.5_	City ground-level fine particulate matter	The Atmospheric Composition Analysis Group in Dalhousie University
CZ	City size	Urban population	China City Statistical Yearbook
PGDP	GDP per capita	GDP per capita	China City Statistical Yearbook
EC	Energy Consumption	The amount of liquefied petroleum gas supply	China City Statistical Yearbook
ES	Economic Structure	Secondary industry output/GDP	China City Statistical Yearbook
TO	Trade Openness	The amount of foreign capital actually utilized	China City Statistical Yearbook
PE	Public Expenditure	Fiscal spending	China City Statistical Yearbook

**Table 3:** Variable descriptive statistics.

Variable	Mean	Standard Deviation	Min	Max
lnPM	3.56	0.47	1.55	4.46
lnCZ	4.69	0.82	2.76	7.79
lnPGDP	10.23	0.60	8.62	12.79
lnEC	9.25	1.50	1.39	13.90
lnES	3.89	0.22	2.77	4.44
lnTO	11.67	2.01	0.00	16.55
lnPE	14.27	0.78	12.05	17.77

**Table 4:** Correlation coefficients.

	lnPM	lnCZ	lnPGDP	lnEC	lnES	lnTO	lnPE
lnPM	1						
lnCZ	0.266 ***	1					
lnPGDP	0.007	0.441 ***	1				
lnEC	0.155 ***	−0.126 ***	0.264 ***	1			
lnES	0.374 ***	0.523 ***	0.479 ***	0.087 ***	1		
lnTO	0.220 ***	0.710 ***	0.714 ***	0.048 **	0.670 ***	1	
lnPE	0.156 ***	0.601 ***	0.370 ***	−0.101 ***	0.500 ***	0.543 ***	1

Note: *** indicates significance at the 1% level. ** indicates significance at the 5% level.

**Table 5:** Empirical results of the static panel model.

Independent Variable	lnPM				
	(1)	(2)	(3)	(4)	(5)
lnCZ	−1.757 ***	−1.738 ***	−1.714 ***	−1.751 ***	−1.699 ***
	(−3.52)	(−3.46)	(−3.59)	(−3.69)	(−3.42)
${\left(\ln CZ\right)}^{2}$	0.340 ***	0.336 ***	0.333 ***	0.341 ***	0.330 ***
	(3.39)	(3.33)	(3.44)	(3.54)	(3.27)
${\left(\ln CZ\right)}^{3}$	−0.022 ***	−0.021 ***	−0.022 ***	−0.022 ***	−0.021 ***
	(−3.29)	(−3.21)	(−3.33)	(−3.45)	(−3.15)
lnPGDP	−0.731 ***	−0.710 ***	−0.613 ***	−0.596 ***	−0.421 **
	(−5.82)	(−5.69)	(−4.36)	(−4.15)	(−2.58)
${\left(\ln PGDP\right)}^{2}$	0.029 ***	0.028 ***	0.024 ***	0.023 ***	0.016 **
	(4.75)	(4.60)	(3.46)	(3.25)	(2.17)
lnEC		−0.009 *	−0.008	−0.008	−0.009 *
		(−1.76)	(−1.53)	(−1.59)	(−1.69)
lnES			−0.066	−0.085 *	−0.111 **
			(−1.44)	(−1.79)	(−2.38)
lnTO				0.007 **	0.007 **
				(2.36)	(2.43)
lnPE					−0.034 *
					(−1.93)
cons	10.960 ***	10.913 ***	10.621 ***	10.600 ***	9.976 ***
	(10.69)	(10.60)	(10.56)	(10.53)	(9.30)
*N*	2389	2389	2389	2389	2389
R-squared	0.122	0.124	0.126	0.129	0.132
F	54.68 ***	44.76 ***	42.07 ***	38.48 ***	34.76 ***

Note: The t statistics are presented in parentheses. *** indicates significance at the 1% level. ** indicates significance at the 5% level. * indicates significance at the 10% level.

**Table 6:** Empirical results of the static panel model for 2007–2011.

Independent Variable	lnPM (2007–2011)				
	(1)	(2)	(3)	(4)	(5)
lnCZ	−1.579 *	−1.541 *	−1.573 *	−1.473 *	−1.506 *
	(−1.97)	(–1.88)	(−1.91)	(−1.75)	(−1.80)
${\left(\ln CZ\right)}^{2}$	0.332 **	0.326 *	0.332 **	0.311 *	0.317 *
	(2.03)	(1.94)	(1.97)	(1.81)	(1.85)
${\left(\ln CZ\right)}^{3}$	−0.023 **	−0.023 **	−0.023 **	−0.022 *	−0.022 *
	(−2.09)	(–2.01)	(−2.04)	(−1.87)	(−1.90)
lnPGDP	−0.979 ***	−0.683 **	−0.704 **	−0.536 *	−0.511 *
	(−3.97)	(−2.55)	(−2.56)	(−1.75)	(−1.68)
${\left(\ln PGDP\right)}^{2}$	0.043 ***	0.029 **	0.030 **	0.024 *	0.022 *
	(3.56)	(2.31)	(2.33)	(1.69)	(1.76)
lnEC		−0.165 *	−0.166 *	−0.167 *	−0.165 *
		(−1.87)	(−1.88)	(−1.93)	(−1.96)
lnES			−0.003	−0.004	−0.004
			(−0.45)	(−0.60)	(−0.59)
lnTO				0.0240	0.025
				(1.21)	(1.28)
lnPE					−0.015 ***
					(−2.65)
cons	11.571 ***	10.532 ***	10.667 ***	9.787 ***	9.866 ***
	(5.82)	(5.29)	(5.26)	(4.38)	(4.42)
*N*	1160	1160	1160	1160	1160
R-squared	0.045	0.05	0.05	0.052	0.056
F	11.12 ***	9.231 ***	7.903 ***	7.893 ***	7.603 ***

Note: The t statistics are presented in parentheses. *** indicates significance at the 1% level. ** indicates significance at the 5% level. * indicates significance at the 10% level.

**Table 7:** Empirical results of the static panel model for 2012–2016.

Independent Variable	lnPM (2012–2016)				
	(1)	(2)	(3)	(4)	(5)
lnCZ	−2.468 ***	−2.592 ***	−2.588 ***	−2.378 ***	−2.378 ***
	(−4.04)	(−4.78)	(−4.76)	(−4.38)	(−4.35)
${\left(\ln CZ\right)}^{2}$	0.487 ***	0.506 ***	0.506 ***	0.454 ***	0.454 ***
	(3.84)	(4.39)	(4.38)	(3.91)	(3.88)
${\left(\ln CZ\right)}^{3}$	−0.032 ***	−0.033 ***	−0.033 ***	−0.028 ***	−0.028 ***
	(−3.69)	(−4.01)	(−4.01)	(−3.46)	(−3.44)
lnPGDP	−1.789 ***	−1.604 ***	−1.603 ***	−0.662 **	−0.661 **
	(−6.95)	(−5.71)	(−5.67)	(−2.01)	(−2.01)
${\left(\ln PGDP\right)}^{2}$	0.074 ***	0.065 ***	0.065 ***	0.025 *	0.025 *
	(6.27)	(4.96)	(4.91)	(1.68)	(1.68)
lnEC		−0.296 ***	−0.284 ***	−0.280 ***	−0.282 ***
		(−4.27)	(−3.94)	(−4.01)	(−3.93)
lnES			−0.002	−0.006	−0.006
			(−0.51)	(−1.38)	(−1.39)
lnTO				0.190 ***	0.190 ***
				(4.70)	(4.66)
lnPE					−0.002
					(−0.17)
cons	18.289 ***	16.375 ***	16.385 ***	13.281 ***	13.298 ***
	(11.25)	(9.73)	(9.73)	(7.60)	(7.57)
*N*	979	979	979	979	979
R-squared	0.127	0.158	0.158	0.200	0.200
F	36.99 ***	37.49 ***	32.51 ***	33.37 ***	29.69 ***

Note: The t statistics are in presented parentheses. *** indicates significance at 1% level. ** indicates significance at 5% level. * indicates significance at 10% level.

**Table 8:** Empirical results of the dynamic panel model.

Independent Variable	lnPM				
	(1)	(2)	(3)	(4)	(5)
$\ln PM_{t-1}$	0.504 ***	0.504 ***	0.520 ***	0.511 ***	0.522 ***
	(90.75)	(114.17)	(204.25)	(290.45)	(166.22)
lnCZ	−0.852 ***	−1.326 ***	−1.157 ***	−0.884 ***	−0.634 ***
	(−10.44)	(−18.47)	(−31.96)	(−18.62)	(−11.79)
${\left(\ln CZ\right)}^{2}$	0.172 ***	0.269 ***	0.237 ***	0.185 ***	0.136 ***
	(11.61)	(20.14)	(33.80)	(19.57)	(12.85)
${\left(\ln CZ\right)}^{3}$	−0.011 ***	−0.017 ***	−0.015 ***	−0.012 ***	−0.009 ***
	(−12.61)	(−21.05)	(−33.95)	(−19.87)	(−12.94)
lnPGDP	−0.556 ***	−0.568 ***	−0.603 ***	−0.532 ***	−0.511 ***
	(−16.65)	(−15.72)	(−34.61)	(−47.21)	(−17.76)
${\left(\ln PGDP\right)}^{2}$	0.026 ***	0.027 ***	0.029 ***	0.025 ***	0.024 ***
	(16.80)	(16.20)	(34.98)	(45.75)	(17.20)
lnEC		−0.034 ***	−0.031 ***	−0.027 ***	−0.022 ***
		(−18.45)	(−23.74)	(−30.20)	(−29.51)
lnES			0.046 ***	0.018 ***	0.003
			(11.85)	(6.18)	(0.78)
lnTO				0.007 ***	0.007 ***
				(34.04)	(34.43)
lnPE					−0.023 ***
					(−24.44)
cons	4.565 ***	5.643 ***	5.352 ***	4.528 ***	4.279 ***
	(23.19)	(25.49)	(51.96)	(51.08)	(23.96)
*N*	1782	1782	1782	1782	1782
Hansen test	245.2	252.1	258.7	259.4	257.7
	[0.004]	[0.088]	[0.458]	[0.909]	[0.998]
AR (1)	−10.21	−10.17	−10.22	−10.20	−10.25
	[0.000]	[0.000]	[0.000]	[0.000]	[0.000]
AR (2)	−0.154	−0.0560	0.323	0.0380	0.255
	[0.878]	[0.955]	[0.746]	[0.970]	[0.799]

Note: The t statistics are presented in parentheses. *p*-values are presented in square brackets. *** indicates significance at the 1% level.

**Table 9:** Regional empirical results.

Independent Variable	lnPM		
	Eastern	Central	Western
lnCZ	−0.968 *	−0.696 ***	−0.139 ***
	(−1.83)	(−2.74)	(−2.86)
${\left(\ln CZ\right)}^{2}$	0.192 *	0.071 **	
	(1.84)	(2.47)	
${\left(\ln CZ\right)}^{3}$	−0.012 *		
	(−1.81)		
lnPGDP	0.509 *	0.001	−1.214 ***
	(1.88)	(0.00)	(−3.33)
${\left(\ln PGDP\right)}^{2}$	−0.02	−0.009	0.049 ***
	(−1.64)	(−0.49)	(2.71)
lnEC	–0.009	–0.004	−0.020 **
	(−1.47)	(−0.52)	(−2.39)
lnES	−0.190 ***	−0.248 ***	0.213 ***
	(−3.92)	(−4.88)	(3.22)
lnTO	0.003	0.008	0.005
	(0.60)	(1.36)	(1.49)
lnPE	−0.149 ***	0.023	0.042
	(−7.79)	(0.83)	(1.35)
cons	5.131 ***	6.781 ***	9.761 ***
	(3.15)	(4.04)	(5.49)
*N*	906	916	567
R-squared	0.144	0.149	0.233
F	14.88 ***	17.67 ***	20.87 ***

Note: The t statistics are presented in parentheses. *** indicates significance at the 1% level. ** indicates significance at the 5% level. * indicates significance at the 10% level.

## References

[1] Han L. (2018). Relationship between urbanization and urban air quality: An insight on fine particulate dynamics in China. Prog. Geogr..

[2] Xie Y., Dai H., Dong H., Hanaoka T., Masui T. (2016). Economic impacts from PM_2.5_ pollution-related health effects in China: A provincial-level analysis. Environ. Sci. Technol..

[3] Tai A.P.K., Mickley L.J., Jacob D.J., Leibensperger E.M., Zhang L., Fisher J.A., Pye H.O.T. (2012). Meteorological modes of variability for fine particulate matter (PM_2.5_) air quality in the united states: Implications for PM_2.5_ sensitivity to climate change. Atmos. Chem. Phys..

[4] Brook R.D., Rajagopalan S., Pope Iii C.A., Brook J.R., Bhatnagar A., Diez-Roux A.V., Holguin F., Hong Y., Luepker R.V., Mittleman M.A. (2010). Particulate matter air pollution and cardiovascular disease: An update to the scientific statement from the American heart association. Circulation.

[5] Pope Iii C.A., Ezzati M., Dockery D.W. (2009). Fine-particulate air pollution and life expectancy in the united states. N. Engl. J. Med..

[6] Landrigan P.J., Fuller R., Acosta N.J.R., Adeyi O., Arnold R., Baldé A.B., Bertollini R., Bose-O’Reilly S., Boufford J.I., Breysse P.N. (2018). The lancet commission on pollution and health. Lancet.

[7] Camagni R., Capello R., Caragliu A. (2013). One or infinite optimal city sizes? In search of an equilibrium size for cities. Ann. Reg. Sci..

[8] Rui-Jun D. (2013). On urban scale and its influencing factors in China: Empirical evidence from 284 cities with the prefecture level and above. J. Financ. Econ..

[9] Xiaolu W. (2010). Urbanization path and city scale in China: An economic analysis. Econ. Res. J..

[10] Grossman G.M., Krueger A.B. (1991). Environmental Impacts of a North American Free Trade Agreement.

[11] Al-Mulali U., Weng-Wai C., Sheau-Ting L., Mohammed A.H. (2015). Investigating the Environmental Kuznets Curve (EKC) Hypothesis by Utilizing the Ecological Footprint as an Indicator of Environmental Degradation. Ecol. Indic..

[12] Ahmad N., Du L., Lu J., Wang J., Li H.-Z., Hashmi M.Z. (2017). Modelling the CO_2_ emissions and economic growth in croatia: Is there any Environmental Kuznets Curve?. Energy.

[13] Wang Y., Han R., Kubota J. (2016). Is there an environmental kuznets curve for SO_2_ emissions? A semi-parametric panel data analysis for China. Renew. Sustain. Energy Rev..

[14] Li T., Wang Y., Zhao D. (2016). Environmental Kuznets Curve in China: New evidence from dynamic panel analysis. Energy Policy.

[15] Han L., Zhou W., Li W. (2018). Growing urbanization and the impact on fine particulate matter (PM_2.5_) dynamics. Sustainability.

[16] Shukla V., Parikh K. (1992). The environmental consequences of urban growth: Cross-national perspectives on economic development, air pollution, and city size. Urban Geogr..

[17] Han L., Zhou W., Li W., Li L. (2014). Impact of urbanization level on urban air quality: A case of fine particles (PM_2.5_) in chinese cities. Environ. Pollut..

[18] Oliveira E.A., Andrade S.J., Makse H.A. (2015). Large cities are less green. Sci. Rep..

[19] Cole M.A., Neumayer E. (2004). Examining the impact of demographic factors on air pollution. Popul. Environ..

[20] Cramer J.C. (2002). Population growth and local air pollution: Methods, models, and results. Popul. Dev. Rev..

[21] Zhang C., Tan Z. (2016). The relationships between population factors and China’s carbon emissions: Does population aging matter?. Renew. Sustain. Energy Rev..

[22] Liddle B. (2015). What are the carbon emissions elasticities for income and population? Bridging stirpat and EKC via robust heterogeneous panel estimates. Glob. Environ. Chang..

[23] Zhou Y., Liu Y. (2016). Does population have a larger impact on carbon dioxide emissions than income? Evidence from a cross-regional panel analysis in China. Appl. Energy.

[24] Lantz V., Feng Q. (2006). Assessing income, population, and technology impacts on co2 emissions in Canada: Where’s the EKC?. Ecol. Econ..

[25] Loomis D., Grosse Y., Lauby-Secretan B., El Ghissassi F., Bouvard V., Benbrahim-Tallaa L., Guha N., Baan R., Mattock H., Straif K. (2013). The carcinogenicity of outdoor air pollution. Lancet Oncol..

[26] Lamsal L.N., Martin R.V., Parrish D.D., Krotkov N.A. (2013). Scaling relationship for NO_2_ pollution and urban population size: A satellite perspective. Environ. Sci. Technol..

[27] Ehrlich P.R., Holdren J.P. (1971). Impact of population growth. Science.

[28] Yu X., Geng Y., Dong H., Ulgiati S., Liu Z., Liu Z., Ma Z., Tian X., Sun L. (2016). Sustainability assessment of one industrial region: A combined method of emergy analysis and ipat (human impact population affluence technology). Energy.

[29] Kaya Y. (1989). Impact of Carbon Dioxide Emission Control on GNP Growth: Interpretation of Proposed Scenarios.

[30] Waggoner P.E., Ausubel J.H. (2002). A framework for sustainability science: A renovated ipat identity. Proc. Natl. Acad. Sci. USA.

[31] Schulze P.C. (2002). I = PBAT. Ecol. Econ..

[32] Xu Z., Cheng G., Qiu G. (2005). Impacts identity of sustainability assessment. Acta Geogr. Sin..

[33] York R., Rosa E.A., Dietz T. (2003). Stirpat, ipat and impact: Analytic tools for unpacking the driving forces of environmental impacts. Ecol. Econ..

[34] Dietz T., Rosa E.A. (1994). Rethinking the environmental impacts of population, affluence and technology. Hum. Ecol. Rev..

[35] Shahbaz M., Loganathan N., Muzaffar A.T., Ahmed K., Jabran M.A. (2016). How urbanization affects co2 emissions in malaysia? The application of stirpat model. Renew. Sustain. Energy Rev..

[36] Chai J., Liang T., Lai K.K., Zhang Z.G., Wang S. (2018). The future natural gas consumption in China: Based on the lmdi-stirpat-plsr framework and scenario analysis. Energy Policy.

[37] Hua Y., Xie R., Su Y. (2018). Fiscal spending and air pollution in chinese cities: Identifying composition and technique effects. China Econ. Rev..

[38] Shao S., Li X., Cao J.H., Yang L.L. (2016). China’s economic policy choices for governing smog pollution based on spatial spillover effects. Econ. Res..

[39] Lin S., Zhao D., Marinova D. (2009). Analysis of the environmental impact of China based on stirpat model. Environ. Impact Assess. Rev..

[40] Poumanyvong P., Kaneko S. (2010). Does urbanization lead to less energy use and lower co2 emissions? A cross-country analysis. Ecol. Econ..

[41] Halkos G.E., Paizanos E.A. (2016). The effects of fiscal policy on CO_2_ emissions: Evidence from the USA. Energy Policy.

[42] Van Donkelaar A., Martin R.V., Brauer M., Kahn R., Levy R., Verduzco C., Villeneuve P.J. (2010). Global estimates of ambient fine particulate matter concentrations from satellite-based aerosol optical depth: Development and application. Environ. Health Perspect..

[43] Xu B., Lin B. (2018). What cause large regional differences in PM_2.5_ pollutions in China? Evidence from quantile regression model. J. Clean. Prod..

[44] Wu J., Zheng H., Zhe F., Xie W., Song J. (2018). Study on the relationship between urbanization and fine particulate matter (PM_2.5_) concentration and its implication in China. J. Clean. Prod..

